# Outcomes of Combined All-Inside With Inside-Out or Outside-In Versus Inside-Out Meniscal Repair Techniques for Bucket-Handle Meniscal Tear

**DOI:** 10.7759/cureus.101443

**Published:** 2026-01-13

**Authors:** Mohamed Shakshak, Sherif Elghazaly, Hossam Diab, Amr H Ahmed, Mahmoud Abdelwahab

**Affiliations:** 1 Trauma and Orthopaedics, Ain Shams University, Cairo, EGY; 2 Trauma and Orthopaedics, Royal Berkshire NHS Foundation Trust, Reading, GBR

**Keywords:** all-inside technique, bucket-handle tear, inside-out, meniscal repair, meniscus outside-in

## Abstract

Bucket-handle meniscal tears (BHMT) are frequently encountered in young, physically active individuals and pose a unique clinical challenge due to their complex morphology and biomechanical implications. The inside-out meniscal repair technique has been regarded as the gold standard due to its robust fixation strength and reproducibility. However, advancements in arthroscopic technology have led to the development of all-inside and hybrid repair techniques, offering less invasive alternatives with comparable biomechanical outcomes and reduced surgical morbidity and time. The purpose of this study is to compare the short-term functional outcomes of different repair techniques of BHMT, comparing the inside-out meniscal repair technique as a gold standard with combined all-inside with outside-in and all-inside with inside-out hybrid techniques. During the period from 2019 to 2024, this prospective randomized controlled study evaluated the functional outcomes of 45 patients who underwent arthroscopic repair of BHMT using either the inside-out meniscal repair technique, combined all-inside with outside-in hybrid technique, or combined all-inside with inside-out hybrid technique. Patients were assessed preoperatively and at three, six, and nine months postoperatively using the Lysholm Knee Scoring Scale. Patients were selected and randomized to one of the three groups. Clinical evaluation was performed using the Lysholm score, as in patients treated with the inside-out technique, the preoperative score was 36.93 ± 12.13 which increased after three months to 75.33 ± 9.54, after six months to 86.57 ± 6.05, and after nine months to 96.07 ± 2.81 indicating fewer symptoms and higher levels of function. Likewise, in patients treated with combined all-inside plus outside-in, the preoperative score was 39.67 ± 10.37, which increased after three months to 77.57 ± 10.6, increasing after six months to 85.78 ± 5.33 and after nine months to 95.89 ± 3.44. In patients treated with combined all-inside plus inside-out, the preoperative score was 42.53 ± 12.94, which increased after three months to 72.13 ± 9.78, increasing after six months to 82.58 ± 8.18 and after nine months to 94.27 ± 4.58. The comparison between the three groups at each time point revealed no statistically significant difference. Except for one case treated with the combined all-inside plus outside-in which was lost to follow-up postoperatively, the mean improvement in Lysholm score was 58.47 ± 13.69 in patients treated with the inside-out technique, 52.43 ± 14.91 in patients treated with combined all-inside with outside-in, and 47.33 ± 15.62 in patients treated with combined all-inside plus inside-out which was not significantly different between the three groups. This finding emphasizes the validity and comparable results of various meniscal repair techniques.

## Introduction

Meniscal injuries are among the most frequently encountered pathologies of the knee joint, particularly in young and physically active individuals [1]. The menisci play a critical role in load transmission, shock absorption, joint stability, lubrication, and proprioception. Loss of meniscal tissue leads to altered knee biomechanics, increased contact pressures, and accelerated articular cartilage degeneration, ultimately predisposing patients to early-onset osteoarthritis. Consequently, meniscal repair has become the preferred treatment strategy over meniscectomy whenever feasible, due to its superior long-term outcomes in preserving knee joint function and delaying degenerative changes [1,2].

Several meniscal repair techniques have evolved over time, each offering distinct advantages and limitations. Historically, open meniscal repair allowed direct visualization and secure fixation, particularly for peripheral tears within the vascularized zone. However, the need for large incisions, prolonged rehabilitation, and a higher risk of neurovascular complications have rendered open repair largely obsolete in the era of modern arthroscopy [2].

The arthroscopic inside-out technique has long been regarded as the gold standard for meniscal repair [2]. It provides strong fixation through vertical mattress sutures, allows accurate suture placement, and has demonstrated excellent healing rates, especially when performed concurrently with anterior cruciate ligament (ACL) reconstruction [2,3]. Nevertheless, its disadvantages include the requirement for accessory posterior incisions, the potential risk of saphenous or common peroneal nerve injury, and an increased likelihood of postoperative stiffness [3,4].

The outside-in technique is particularly effective for anterior horn and mid-body meniscal tears. It is technically simpler and safer in these regions, offering precise suture control with minimal risk to neurovascular structures. However, its application is limited in posterior horn tears, and subcutaneous knot irritation remains a recognized drawback [5].

Advancements in arthroscopic instrumentation have led to the widespread adoption of all-inside meniscal repair techniques. These devices allow minimally invasive repair, reduced operative time, and improved access to posterior horn tears with a lower risk of neurovascular injury [6,7]. Despite these advantages, all-inside techniques are associated with higher costs, device dependency, and specific complications such as chondral damage, implant migration, and foreign body reactions [6,7].

More recently, hybrid repair strategies combining all-inside with either inside-out or outside-in techniques have been introduced. These combined approaches aim to capitalize on the biomechanical strength of sutures while enhancing safety and surgical efficiency. Early biomechanical and clinical studies suggest promising stability and potential cost-effectiveness; however, robust long-term outcome data remain limited [8].

Systematic reviews and meta-analyses indicate that inside-out and all-inside techniques achieve comparable healing rates and functional outcomes. While some studies report superior patient-reported outcomes with all-inside repairs, these may be offset by a higher incidence of device-related complications. Conversely, the inside-out technique is associated with a greater risk of neurovascular injury. Therefore, the choice of meniscal repair technique should be individualized based on tear morphology, anatomical location, patient activity demands, and surgeon expertise. The overarching goal remains the preservation of meniscal tissue to optimize knee function and delay degenerative progression [1,2,6,7,9].

The primary objective of this study was to compare the short-term functional outcomes of conventional inside-out meniscal repair (Group A) with two combined techniques, namely, all-inside plus outside-in (Group B) and all-inside plus inside-out (Group C), in the management of bucket-handle meniscal tears (BHMT). It was hypothesized that combined techniques might provide superior biomechanical stability and, consequently, improved clinical outcomes compared to the traditional inside-out method.

## Materials and methods

Data collection and follow-up

Patients were prospectively enrolled according to strict eligibility criteria to ensure the homogeneity of the study population and to minimize confounding factors.

Inclusion Criteria

Individuals aged between 18 and 55 years, of either sex, who presented with symptomatic mechanical locking of the knee and radiological confirmation of a BHMT on magnetic resonance imaging (MRI) were included.

Exclusion Criteria

Patients were excluded if they demonstrated advanced knee osteoarthritis or significant articular surface injury. Additionally, those with concomitant ligamentous injuries, such as collateral ligament involvement, or with coronal plane deformities, including varus or valgus malalignment, were excluded to avoid bias in the functional outcomes. This selection strategy allowed the study cohort to represent patients with isolated BHMT amenable to repair while excluding confounders that could influence surgical results or long-term prognosis.

**Figure 1:**
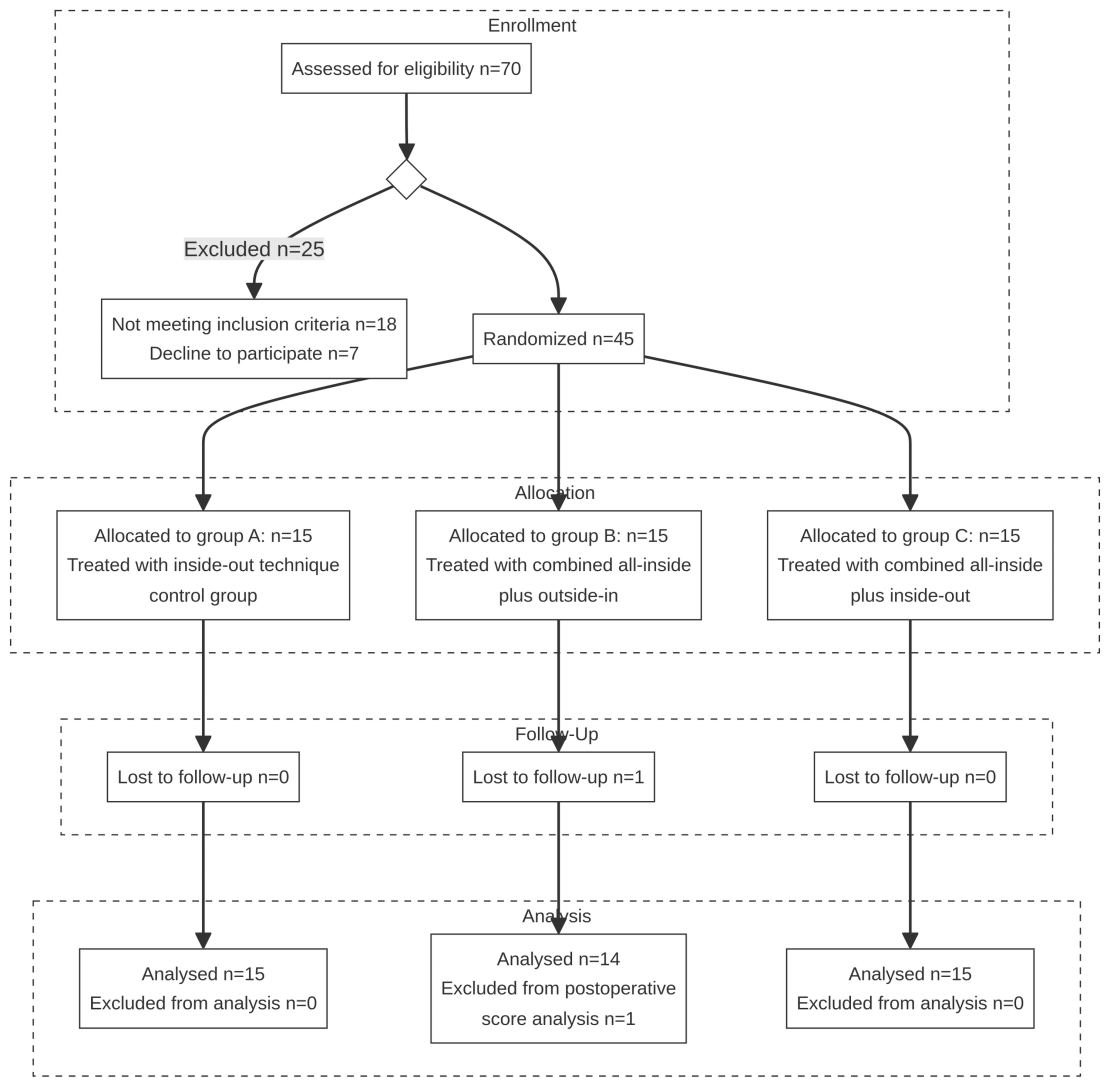
CONSORT flowchart of the progress through the phases of a three-group parallel randomized study CONSORT: Consolidated Standards of Reporting Trials

This prospective randomized controlled study was conducted at Ain Shams University Hospitals and Nasser Institute for Research and Treatment, Cairo, between June 2019 and January 2024 to compare clinical outcomes among three surgical techniques for bucket-handle meniscal repairs. The study initially enrolled 70 cases, 25 of which were excluded due to significant chondral damage or neglected bucket-handle tear or concomitant collateral ligamentous injuries (Figure 1). The study included 45 knees from patients aged 18-55 years presenting with mechanical locking (loss of full extension), restricted range of motion (loss of flexion), and MRI-confirmed bucket-handle tears with no significant chondral damage or associated collateral ligament injuries.

Randomization

Participants who fulfilled the inclusion criteria and provided consent were enrolled in the study. Eligible patients were then randomized into three equal groups using the sealed envelope method, which ensured allocation concealment and minimized selection bias. Group A served as the control group and underwent conventional inside-out meniscal repair. Group B received a hybrid repair combining all-inside with outside-in techniques, while Group C underwent a hybrid repair combining all-inside with inside-out techniques. This randomization strategy was designed to distribute potential confounding factors evenly across the study arms, thereby allowing for a reliable comparison of surgical outcomes among the three techniques.

Evaluations

Preoperative evaluation included clinical history, physical and orthopedic examination, standing radiographs, MRI confirmation, and baseline Lysholm scoring. Postoperative management consisted of antibiotic prophylaxis, analgesia, and follow-up at three, six, and nine months with clinical evaluation and repeated Lysholm scoring.

Statistical analyses were performed using IBM SPSS Statistics for Windows, V. 28.0 (IBM Corp., Armonk, NY, USA), employing one-way analysis of variance (ANOVA) for parametric data, chi-squared tests for categorical variables, and linear regression to identify outcome predictors. A p-value of <0.05 was considered statistically significant.

The present study included 45 young adult patients (29 males and 16 females) diagnosed with BHMT, who were randomly allocated into three equal groups. The reference group underwent inside-out meniscal repair using a Clancy needle technique. The remaining two groups, as described in the Methods section, comprised 15 patients treated with a combined all-inside plus inside-out repair and 15 patients treated with a combined all-inside plus outside-in repair. All-inside meniscal fixation was performed using either the FAST-FIX device (Smith & Nephew plc, Watford, Hertfordshire, UK) or the Cinch meniscal repair system (Arthrex, Inc., Naples, FL, USA).

Surgical procedures

After randomization and obtaining informed consent, all patients underwent diagnostic arthroscopy. This direct visualization of the tear and intra-articular space provided a definitive understanding of the pathology.

On the operation table, the patient lies supine. A tourniquet is applied to the upper thigh. To enable access to the medial and lateral compartments with valgus pressure and the figure-four posture, respectively, a post for the lateral thigh is necessary.

The anteromedial and anterolateral standard portals are formed during patient preparation and drapement. Typically, a 30-degree, 4-mm arthroscope is utilized. Due to its reduced diameter, a 2.9-mm arthroscope is also sometimes utilized to increase posterior vision. It is possible to see most rips via the anterolateral portal; however, switching to the anteromedial portal may provide a better view of the front part of the lateral meniscus.

Furthermore, in knees with tight medial compartments, a partial medial collateral ligament (MCL) release (pie-crusting) may be necessary to provide enough visibility. This may be achieved by a spinal needle from the "outside-in" trephination of the ligament origin. Upon completion of the diagnostic arthroscopy, the meniscal tear will become the primary focus of treatment. To determine the exact nature of the rip, a small probe is inserted into the joint.

The probe's tip may be carefully inserted into the tear to see how severe it is. The menisci may also have tears in their horizontal cleavage that wouldn't be seen unless pressure was applied to the top and bottom surfaces.

Meniscal tear preparation is analogous to bone fracture therapy for non-union. Evaluation of vascularity, tear reduction, and application of stable fixation are important. Any part of the process that doesn't go well might affect how well the repair turns out in the end. A little shaver (without suction) or a small rasp may be used to debride the tear edges. Polishing these edges not only improves visibility but also stimulates a healing response that makes the repair stronger.

The minimum and maximum number of sutures used in a meniscal repair were two and six, respectively.

Rehabilitation protocol

In the early phase (0-6 weeks), rehabilitation focuses on protecting the repair by limiting knee loading and motion, typically using a brace locked in extension with touch-down or partial weight-bearing on crutches, restricting knee flexion to 0-90°, and avoiding active hamstring contraction, squatting, twisting, or pivoting while prioritizing pain and swelling control and early quadriceps activation. From approximately six to 12 weeks, patients progressively advance to full weight-bearing, wean off crutches and brace as tolerated, restore full pain-free range of motion, and begin controlled strengthening and proprioceptive exercises while still avoiding deep flexion and high-stress activities. In the later phase (beyond 12 weeks), rehabilitation emphasizes the normalization of gait, advanced strengthening, endurance, balance, and proprioception, with a gradual, criteria-based return to running and sport-specific activities, ensuring that progression is guided by clinical recovery rather than time alone.

The study protocol was reviewed and approved by the Faculty of Medicine, Ain Shams University Research Ethics Committee (FMASU REC), in September 2019 (approval number: FMASU MD 273/2019). Prior to inclusion, each participant received detailed information regarding the study objectives, procedures, potential risks, and expected benefits. Written informed consent was obtained from all participants, ensuring respect for patient autonomy and the principles of voluntary participation. Confidentiality of patient data was maintained throughout the study, and no identifying information was disclosed in the reporting of results.

The FMASU REC is organized and operated according to the guidelines of the International Council for Harmonization (ICH) Anesthesiology and the Islamic Organization for Medical Sciences (IOMS), the United States Office for Human Research Protections, and the United States Code of Federal Regulations and operates under Federal Wide Assurance No. FWA 00017585. The REC does not declare the names of its members according to the University and the REC's standard operating procedures.

## Results

**Figure 2:**
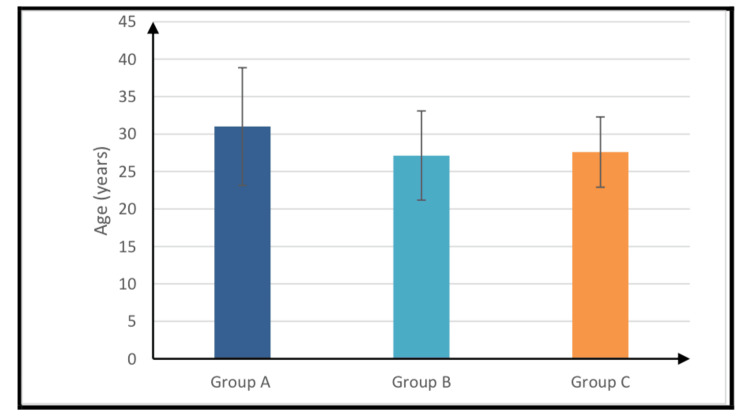
Mean age of the studied groups

**Figure 3:**
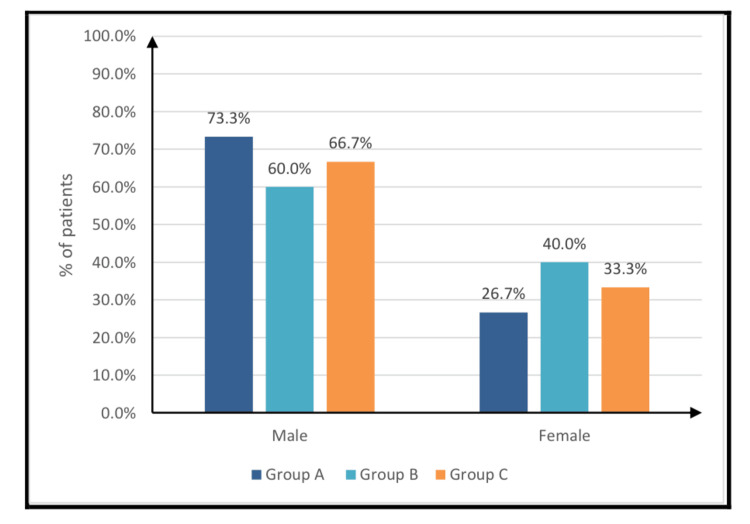
Sex distribution of the studied groups

As illustrated in Table 1, no statistically significant differences were observed among the three study groups with respect to age or sex distribution. These demographic characteristics are further detailed in Figure 2 and Figure 3.

**Table 1:** Demographic data of the studied groups Data are presented as frequency (%) unless otherwise mentioned. Statistical significance is defined at p<0.05.

	Group A (n = 15)	Group B (n = 15)	Group C (n = 15)	P-value
Age (years)				
Mean ± SD	31 ± 7.86	27.13 ± 5.95	27.6 ± 4.69	0.198
Range	18-47	20-39	18-37	
Sex				
Male	11 (73.3%)	9 (60%)	10 (66.7%)	0.741
Female	4 (26.7%)	6 (40%)	5 (33.3%)	

There was no statistically significant difference between the three groups in terms of diagnosis, injury side, or the performed procedures as summarized in Table 2.

**Table 2:** Diagnosis and procedures performed on the studied groups Data are presented as frequency (%) unless otherwise mentioned. Statistical significance is defined at p<0.05. MM: medial meniscus; LM: lateral meniscus; ACL: anterior cruciate ligament

	Group A (n = 15)	Group B (n = 15)	Group C (n = 15)	P-value
Diagnosis				
MM bucket-handle tear	5 (33.3%)	3 (20%)	9 (60%)	0.067
LM bucket-handle tear	10 (66.7%)	9 (60%)	4 (26.7%)	
ACL + MM and/or LM	0 (0%)	3 (20%)	2 (13.3%)	
Side				
Right MM bucket-handle tear	4 (26.7%)	5 (33.3%)	5 (33.3%)	0.605
Left MM bucket-handle tear	1 (6.7%)	1 (6.7%)	4 (26.7%)	
Right LM bucket-handle tear	3 (20%)	6 (40%)	2 (13.3%)	
Left LM bucket-handle tear	7 (46.7%)	3 (20%)	4 (26.7%)	
Procedure				
Meniscal repair	15 (100%)	12 (80%)	13 (86.7%)	0.1
ACL reconstruction + meniscal repair	0 (0%)	3 (20%)	2 (13.3%)	

Clinical outcomes were evaluated using the Lysholm score. In patients treated with the inside-out technique, the mean preoperative score was 36.93 ± 12.13. This score increased to 75.33 ± 9.54 at three months, 86.57 ± 6.05 at six months, and 96.07 ± 2.81 at nine months, indicating a progressive improvement in function and reduction of symptoms. Similarly, the group treated with the combined all-inside and outside-in technique demonstrated an increase from a preoperative score of 39.67 ± 10.37 to 77.57 ± 10.6 at three months, 85.78 ± 5.33 at six months, and 95.89 ± 3.44 at nine months. In the group treated with the combined all-inside and inside-out technique, scores improved from 42.53 ± 12.94 preoperatively to 72.13 ± 9.78 at three months, 82.58 ± 8.18 at six months, and 94.27 ± 4.58 at nine months.

**Figure 4:**
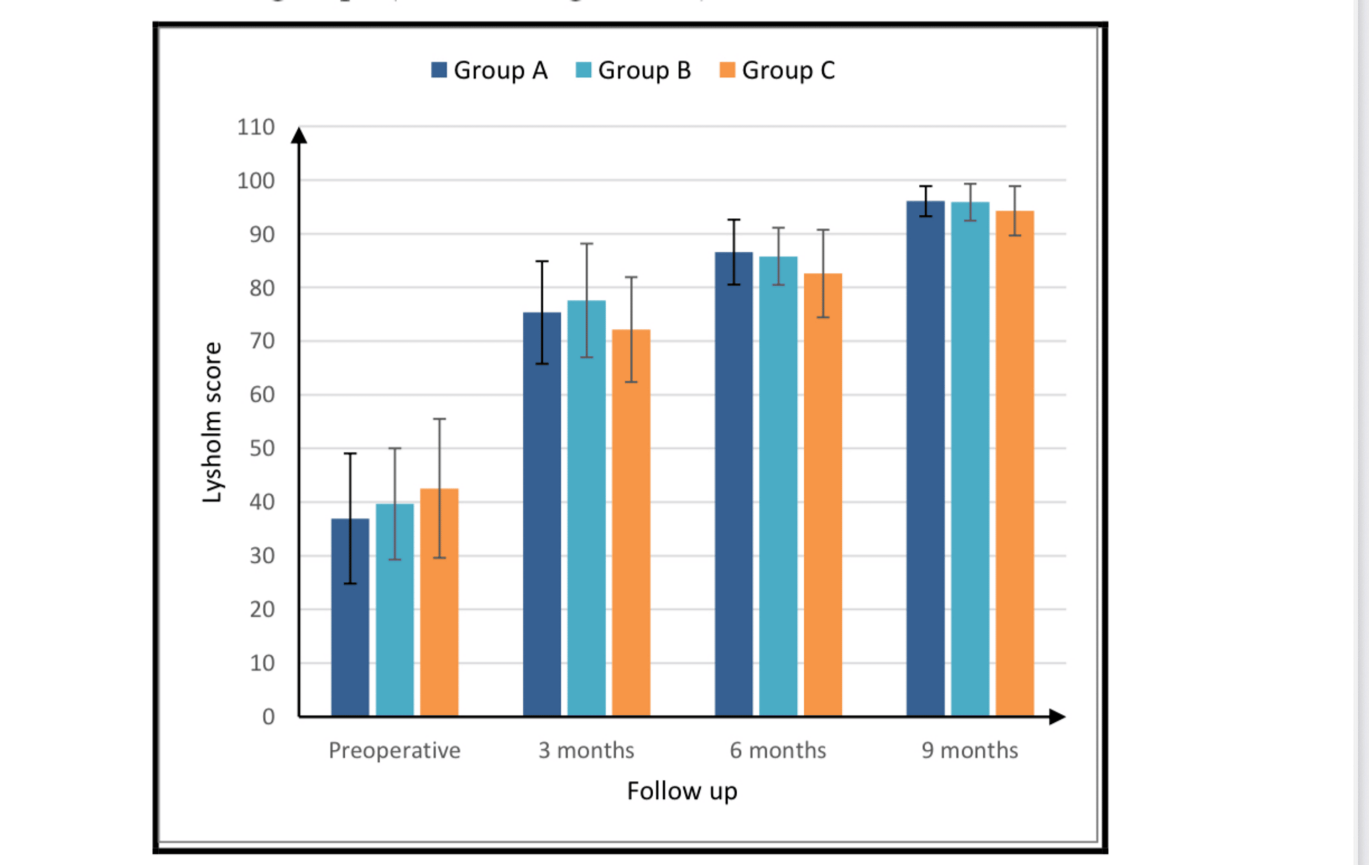
Lysholm score evaluation pre- and postoperatively

Intergroup comparisons at each postoperative time point demonstrated no statistically significant differences in Lysholm scores. One patient in the combined all-inside and outside-in group was lost to follow-up. The mean improvement in Lysholm score (final minus preoperative) was 58.47 ± 13.69 in the inside-out group, 52.43 ± 14.91 in the combined all-inside and outside-in group, and 47.33 ± 15.62 in the combined all-inside and inside-out group. No statistically significant difference in mean improvement was observed among the three groups (Table 3 and Figure 4).

**Table 3:** Lysholm score evaluation pre- and postoperatively

	Group A (n = 15)	Group B (n = 15)	Group C (n = 15)	P-value
Lysholm score				
Preoperative	36.93 ± 12.13	39.67 ± 10.37	42.53 ± 12.94	0.441
3 months	75.33 ± 9.54	77.57 ± 10.6	72.13 ± 9.78	0.345
6 months	86.57 ± 6.05	85.78 ± 5.33	82.58 ± 8.18	0.308
9 months	96.07 ± 2.81	95.89 ± 3.44	94.27 ± 4.58	0.437
Improvement	58.47 ± 13.69	52.43 ± 14.91	47.33 ± 15.62	0.131

As detailed in Table 4, the incidence of postoperative complications did not differ significantly among the three treatment groups. A single case of knee instability (giving way) was reported in the combined all-inside and outside-in group.

**Table 4:** Complications of the studied groups

	Group A (n = 15)	Group B (n = 15)	Group C (n = 15)	P-value
No complications	15 (100%)	14 (93.3%)	14 (93.3%)	0.35
Giving way	0 (0%)	1 (6.7%)	0 (0%)	
Limited extension/improved with physical rehabilitation	0 (0%)	0 (0%)	1 (6.7%)	

**Figure 5:**
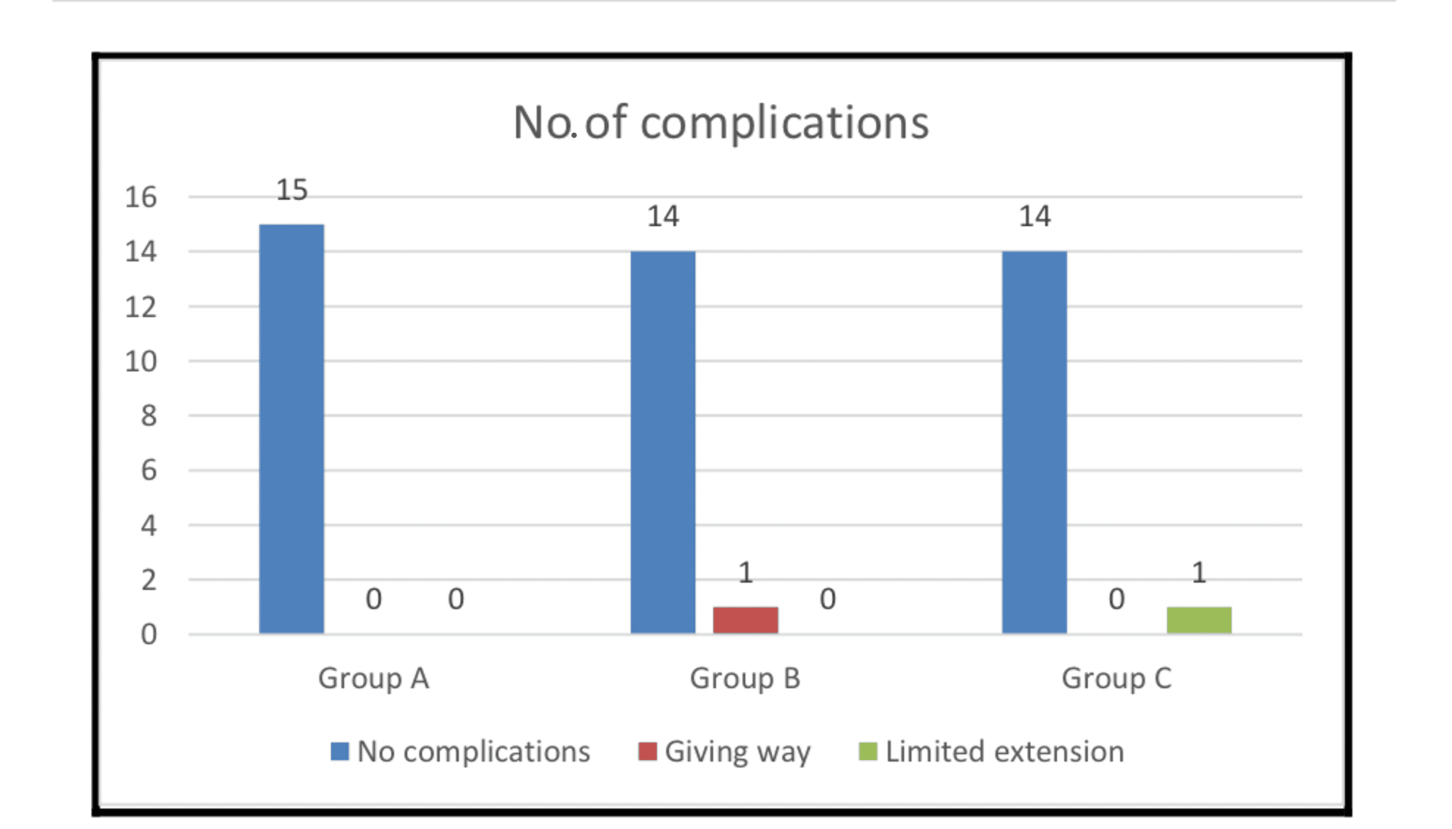
Number of complications in each group

Similarly, one case of limited knee extension, which subsequently resolved with physiotherapy, was observed in the combined all-inside and inside-out group. The overall complication profile is further illustrated in Figure 5.

A linear regression model was employed to identify factors associated with the improvement in Lysholm score over time while controlling for the covariates of age, sex, and diagnosis. This analysis compared the performance of the three surgical techniques. In both univariate and multivariate linear regression models, the repair technique was not a statistically significant predictor of improvement in the Lysholm score (Table 5).

**Table 5:** Linear regression model for factors associated with the improvement in Lysholm score over time MM: medial meniscus; LM: lateral meniscus; ACL: anterior cruciate ligament

	Univariate			Multivariable		
	Coefficient	95% CI	P-value	Coefficient	95% CI	P-value
Age (years)	0.21	-0.53 to 0.94	0.573	0.08	-0.70 to 0.85	0.843
Sex						
Male	Ref			Ref		
Female	1.39	-8.44 to 11.22	0.777	1.86	-7.93 to 11.66	0.702
Diagnosis						
MM	Ref			Ref		
LM	6.39	-3.19 to 15.98	0.186	4.26	-6.39 to 14.90	0.423
ACL + MM and/or LM	-8.07	-23.18 to 7.04	0.287	-7.38	-23.59 to 8.84	0.363
Technique of repair						
Inside-out technique	Ref			Ref		
Combined all-inside with outside-in	-6.04	-17.12 to 5.04	0.277	-4.02	-16.31 to 8.27	0.512
Combined all-inside with inside-out	-11.13	-22.02 to -0.25	0.045	-8.31	-20.55 to 3.92	0.177

All statistical analyses were conducted using EasyMedStat® (Neuilly-Sur-Seine, France). Continuous variables are presented as mean ± standard deviation and range and were compared using Student's t-test or the Wilcoxon rank-sum test, as appropriate. Categorical variables were compared using Fisher's exact test. Survival analysis was performed using the Kaplan-Meier method. A p-value of less than 0.05 was considered statistically significant for all analyses.

## Discussion

The primary objective of this study was to compare the short-term functional outcomes of the conventional inside-out meniscal repair technique (Group A) with two combined approaches, that is, all-inside plus outside-in (Group B) and all-inside plus inside-out (Group C), for the treatment of BHMT. Our initial hypothesis posited that the combined techniques might offer a more favorable and biomechanically stronger fixation construct, potentially leading to superior clinical outcomes.

The principal finding of this study was that all three meniscal repair techniques, namely, inside-out, combined all-inside plus outside-in, and combined all-inside plus inside-out, achieved comparable functional outcomes as measured by the Lysholm score, with equivalent clinical failure rates and similar complication profiles over a nine-month follow-up period. Specifically, no statistically significant differences were observed in Lysholm scores among the three groups at three, six, or nine months postoperatively (Figure 4).

Although the inside-out technique demonstrated a numerically greater mean improvement in Lysholm score (58.47 ± 13.69) compared to the combined all-inside plus outside-in (52.43 ± 14.91) and combined all-inside plus inside-out (47.33 ± 15.62) groups, this difference did not reach statistical significance (p = 0.131). Complication rates were similarly low and not significantly different across the groups (Figure 5). These results do not support our initial hypothesis, indicating that the combined techniques, despite their theoretical biomechanical advantages, do not confer superior short-term clinical outcomes compared to the conventional inside-out method. However, their role in zone-specific repair is critical, as in situations where a bucket-handle tear is extended to the anterior horn, using the outside-in repair technique is the convenient solution. Furthermore, if the tear is extended to the posterior horn closer to the root, all-inside is safer and much easier to apply.

The current results suggest that while newer combined techniques offer additional surgical flexibility, the traditional inside-out method remains a reliable gold standard.

Notably, complications were infrequent and did not differ significantly between groups, supporting the safety profile of all techniques evaluated. Regression analysis indicated a trend toward greater improvement with the inside-out technique; however, age, sex, and specific tear location were not significant predictors of outcome in the multivariate analysis. This reinforces the perspective that the choice of surgical technique should be individualized, considering surgeon expertise, tear characteristics, and resource availability.

Meniscal preservation through repair, as opposed to meniscectomy, mitigates the risk of early-onset osteoarthritis, as established by Papalia et al. [10]. Our findings further substantiate that appropriate repair, even in complex BHMT, facilitates a return to function and athletic activity with minimal complication risk.

BHMT are prevalent, particularly among young, active patients, and pose a considerable clinical challenge. In most cases, subtotal meniscectomy is undesirable due to the resultant biomechanical alterations and well-documented poor long-term outcomes. Currently, there is no universally ideal technique for repairing BHMT, as long-term implant survival studies comparing different methods are lacking.

Our results align with previous biomechanical studies demonstrating equivalent fixation properties between modern all-inside implants and traditional inside-out sutures [10,11]. The biomechanical strength of the FAST-FIX device has been shown to be comparable to vertical mattress sutures while maintaining similar load-to-failure characteristics when deployed in a horizontal configuration [10,11].

A review of the literature indicates that three systematic reviews published within the past decade have compared inside-out and all-inside meniscal repairs [12,13]. The systematic reviews by Nepple et al. [12] and Ayeni et al. [13] exclusively included all-inside studies utilizing the meniscal arrow. Grant et al. [14] conducted the only systematic review directly comparing inside-out and all-inside repairs, although the all-inside cohort incorporated both outdated and contemporary devices.

The results of our study are consistent with the conclusion of Grant et al. [14], indicating no significant difference in clinical outcomes between inside-out and all-inside meniscal repairs. In contrast, Lysholm scores were similar between Grant et al. [14] and our study. Grant et al. [14] reported mean Lysholm scores of 87.8 for inside-out repairs and 90.2 for all-inside repairs, whereas our analysis yielded scores of 85.71 ± 3.75 for inside-out, 86.33 ± 3.57 for all-inside plus outside-in, and 86.73 ± 3.55 for all-inside plus inside-out repairs. Many surgeons advocate for all-inside repairs for smaller tears requiring only one or two implants while preferring inside-out repair for larger tears.

We support a preference for inside-out repairs in larger tears, as these were typically stabilized with an average of three sutures, compared to two devices for all-inside repairs. Despite this institutional preference, patients managed exclusively with all-inside techniques during the study period (though not included in this study) were observed to have similar functional outcomes and failure rates.

Study limitations primarily relate to the collection of Lysholm scores, which depended on physiotherapy-led follow-up visits. Postoperative follow-up was coordinated through physiotherapy services, with an open referral pathway back to the soft tissue knee clinic if complications were identified. Recorded complications included slow rehabilitation progress, limited range of motion, and episodes of giving way. None of the cases required surgical reintervention, and all complications resolved with extended physiotherapy.

This study underscores the comparable validity and outcomes of different meniscal repair techniques, which may hold economic implications in resource-constrained environments. Reversion to the gold standard inside-out technique may offer substantial cost savings. Furthermore, the trend toward replacing inside-out and outside-in techniques with all-inside devices may reduce operative time, providing an economic advantage by increasing theatre efficiency and capacity, as operational costs often exceed implant expenses.

Interpretation of the results in the context of existing literature

The consistent improvement in Lysholm scores across all three groups from preoperative baseline to nine months postoperatively (Figure 4) underscores the effectiveness of meniscal repair in restoring knee function and reducing symptoms in patients with bucket-handle tears. This aligns with the broader consensus in orthopedic literature that meniscal repair, when indicated, is a viable and beneficial procedure for preserving knee joint integrity and function [10].

Our finding of no significant difference in clinical outcomes between the inside-out technique and the combined all-inside approaches is consistent with several systematic reviews. Grant et al. [14], in their systematic review comparing inside-out and all-inside repairs, also concluded that there was no significant difference in clinical results. This review, however, included both outdated and modern all-inside meniscal repair devices.

The current study, by specifically utilizing modern all-inside devices such as FAST-FIX or Cinch in the combined groups, suggests that even with contemporary implants, a definitive clinical superiority over inside-out repair for bucket-handle tears may not be evident in the short to medium term. This finding is particularly relevant given the ongoing advancements in all-inside repair technology.

Nepple et al. [12] and Ayeni et al. [13] conducted systematic reviews that primarily included all-inside studies focusing on the meniscal arrow. While these reviews highlighted the evolution and specific outcomes of certain all-inside devices, they did not directly compare combined approaches with inside-out techniques as comprehensively as the current study.

The biomechanical literature has often suggested advantages for newer all-inside devices and specific suture configurations. Post et al. [15] demonstrated that vertical mattress techniques are biomechanically superior to horizontal mattress techniques for suture fixation in meniscal repair. This biomechanical advantage is crucial as it directly impacts the stability of the repair.

Furthermore, studies by Barber et al. [16] and Borden et al. [17] have shown that the biomechanical strength of modern all-inside devices, such as the FAST-FIX, is comparable to traditional vertical mattress sutures, maintaining similar load to failure. While these biomechanical studies suggest that modern all-inside implants offer robust fixation, our clinical findings indicate that this biomechanical equivalence translates to similar, rather than superior, clinical outcomes when compared to the gold standard inside-out technique. This could imply that beyond a certain threshold of fixation strength, additional biomechanical advantages may not yield further clinical benefits or that other biological factors, such as vascularity and cellular response, play a more dominant role in overall healing in the clinical setting.

The low incidence of complications observed in our study, with no statistically significant difference between groups (Figure 5), further supports the safety profile of all three repair strategies. Only one case in Group B (combined all-inside plus outside-in) experienced "giving way," and one case in Group C (combined all-inside plus inside-out) had "limited extension" that improved with physiotherapy. This represents a notable improvement compared to previously reported failure rates in the literature. Grant et al. [14], for instance, reported clinical failure rates of 17% for inside-out and 19% for all-inside repairs in their systematic review. In contrast, our study observed 0% failure rates in the inside-out and combined all-inside with inside-out or outside-in groups during our nine-month follow-up period. This significant reduction in failure rates could be attributed to several factors, including improved implant design, advancements in surgical techniques, more rigorous patient selection, and a collective learning curve in meniscal repair over time. The resolution of the limited extension complication with physiotherapy also highlights the critical role of comprehensive postoperative rehabilitation.

Our linear regression analysis (Table 5) indicated that while the combined all-inside plus inside-out technique showed a statistically significant lower improvement in Lysholm score compared to the inside-out technique in univariate analysis (coefficient: -11.13; 95% CI: -22.02 to -0.25; p = 0.045), this significance was lost in the multivariable analysis when controlling for age, sex, and diagnosis (coefficient: -8.31; 95% CI: -20.55 to 3.92; p = 0.177).

This suggests that patient demographics and tear characteristics, while important for surgical planning, may not be independent predictors of functional outcome once a successful repair is achieved. The lack of significant association in multivariate analysis reinforces the idea that all three techniques, when applied appropriately, can lead to comparable positive outcomes.

Our study's focus on repair techniques for bucket-handle tears, which often involve significant meniscal displacement, aligns with the critical need for tissue preservation. The successful clinical outcomes observed across all groups further support the efficacy of modern repair strategies in maintaining knee homeostasis.

The inherent healing capacity of the meniscus is profoundly influenced by its vascularity. As described by Arnoczky and Warren [18], the perimeniscal capillary network supplies the outer 10-30% of the meniscus (the red-red zone), which possesses significant healing potential. In contrast, the central third (white-white zone) is largely avascular and has a diminished capacity for spontaneous healing [19]. Bucket-handle tears, being longitudinal tears that often displace into the intercondylar notch, typically involve the vascularized periphery, making them amenable to repair. The surgical preparation of the tear edges through debridement and rasping, as detailed in the Methodology, is crucial as it aims to stimulate a healing response by promoting vascular ingrowth and cellular activity, akin to a bone fracture non-union treatment [20].

Beyond vascularity, cellularity and the local biochemical and mechanical environment also play critical roles in meniscal healing. Meniscal cells are responsible for extracellular matrix (ECM) remodeling. However, cellularity declines with age, contributing to reduced healing potential in mature patients [21,22]. Furthermore, inflammatory cytokines like IL-1 and TNF-α can hinder healing by activating catabolic matrix metalloproteinases (MMPs) [23,24].

Conversely, anabolic growth factors promote cell growth and matrix production. Mechanical stimuli, such as dynamic compression, can also influence gene expression and inflammation, highlighting the importance of adequate joint mobility for meniscal repair [25-27]. While our study did not directly incorporate biological adjuncts or explicitly manipulate mechanical loading beyond standard rehabilitation, the successful outcomes observed suggest that current surgical techniques, combined with appropriate patient selection and a structured rehabilitation program, are effective in leveraging the inherent healing capacity of the meniscus.

The finding that all three techniques yield similar functional outcomes and complication rates has significant clinical implications. It suggests that surgeons have flexibility in choosing the repair method based on tear characteristics, surgeon experience, and resource availability, without compromising short-term patient outcomes.

Limitations of the study

Despite the methodological strength of this work as a prospective randomized controlled study, several limitations should be acknowledged when interpreting the findings. Firstly, the relatively small sample size, with 15 knees allocated to each group, may have limited the statistical power of the study to detect subtle yet clinically relevant differences between the repair techniques. Although no statistically significant differences were observed, it is plausible that a larger cohort could reveal more nuanced distinctions in functional or complication-related outcomes.

Secondly, the duration of follow-up was limited to nine months, which primarily reflects short-term functional recovery. While this period is sufficient to assess early postoperative outcomes, it does not allow for the evaluation of long-term consequences such as the progression of osteoarthritic changes, the durability of the meniscal repair, or implant survival. Meniscal healing is a prolonged biological process, and degenerative joint changes often become apparent only after several years; therefore, the true long-term effectiveness of the repair techniques cannot be fully determined from the present data.

Another important limitation is the absence of objective imaging or arthroscopic confirmation of meniscal healing. Outcome assessment relied exclusively on clinical measures, including the Lysholm knee score and recorded complication rates. Although clinical improvement is highly relevant, it does not always correlate with complete anatomical healing. The lack of follow-up MRI or second-look arthroscopy restricts the ability to confirm the structural integrity of the repaired meniscus and may underestimate subclinical failure rates.

Furthermore, the Lysholm score, while widely validated and commonly used in knee outcome research, is a patient-reported measure and therefore inherently subjective. Patient perception of pain, function, and instability may be influenced by psychosocial factors and expectations, potentially introducing response bias into the outcome assessment.

In addition, some heterogeneity existed within the all-inside repair techniques used. Although FAST-FIX and Cinch devices were both employed, the precise distribution of these devices within the combined repair groups was not specified. Despite both being modern, widely accepted systems, subtle differences in biomechanical properties or deployment techniques could have influenced the results and should be considered when interpreting the findings.

Finally, Lysholm score data were collected during physiotherapy follow-up visits. While this approach is practical and reflects routine clinical practice, it introduces potential inter-observer variability, even when a standardized rehabilitation and assessment protocol is followed. This may have contributed to minor inconsistencies in outcome reporting.

Future research directions

Future investigations should prioritize long-term prospective follow-up, with assessment intervals extending to two, five, and 10 years postoperatively. Such studies would allow the evaluation of repair durability, the incidence of osteoarthritis, and the need for revision surgery, thereby providing a more comprehensive understanding of the long-term protective role of different meniscal repair techniques.

Incorporation of objective measures of meniscal healing is also strongly recommended. Serial MRI using validated meniscal healing protocols, or planned second-look arthroscopy where ethically and clinically appropriate, would enable the correlation of clinical outcomes with anatomical repair integrity and enhance the robustness of future studies.

Additionally, formal cost-effectiveness analyses are warranted to compare inside-out, all-inside, and combined repair techniques. These analyses should account for direct costs, including implants, operative time, and hospital resources, as well as indirect costs related to rehabilitation, time off work, complications, and potential re-operations.

Larger multicenter randomized controlled trials would further improve statistical power and external validity, increasing the generalizability of findings across different patient populations and surgical settings. This may also allow the detection of smaller but clinically meaningful differences between techniques.

Emerging biological augmentation strategies represent another promising area for future research. The adjunctive use of platelet-rich plasma, bone marrow aspirate concentrate, stem cells, or growth factors may enhance healing, particularly in the relatively avascular zones of the meniscus, and should be investigated in conjunction with established repair techniques.

Moreover, subgroup analyses based on tear characteristics, such as tear length, stability, chronicity, and the presence of associated ligamentous injuries, may help identify patient- or tear-specific factors that favor one repair technique over another.

Finally, while the Lysholm score remains a valuable outcome measure, future studies should consider incorporating a broader range of patient-reported outcome measures, including the Knee Injury and Osteoarthritis Outcome Score and the International Knee Documentation Committee Subjective Knee Evaluation Form. The use of multiple patient-reported outcome measures would provide a more comprehensive evaluation of knee function, symptoms, and quality of life from the patient's perspective.

## Conclusions

This study provides valuable insights into the contemporary management of BHMT. It demonstrates that both the conventional inside-out repair and the combined all-inside with outside-in or inside-out techniques yield comparable favorable short-term clinical and functional outcomes with low complication rates. Although our initial hypothesis regarding superior outcomes with combined techniques was not substantiated, the study reinforces the inside-out technique as a reliable and effective gold standard. The selection of repair technique should be individualized based on patient-specific factors, tear characteristics, and surgical expertise while maintaining a focus on meniscal preservation to mitigate long-term degenerative changes. Future investigations incorporating extended follow-up durations, objective healing assessments, and comprehensive cost-effectiveness analyses will further elucidate optimal management strategies for this prevalent and clinically significant knee injury.

The limitations of this study include a relatively modest sample size, the absence of long-term follow-up beyond nine months, and the lack of radiographic confirmation of healing. Subsequent studies should incorporate second-look arthroscopy or MRI to evaluate tissue healing, alongside a minimum two-year follow-up period to assess progression toward osteoarthritis.

In conclusion, while all three techniques demonstrated clinical efficacy, the inside-out method remains a benchmark procedure. Combined techniques show promise for specific tear configurations. Surgical decision-making should be guided by patient factors, tear morphology, and surgeon experience, with tissue preservation constituting the fundamental priority.
